# Glymphatic Dysfunction in Children With Type 2 and 3 Spinal Muscular Atrophy

**DOI:** 10.1002/cns.71063

**Published:** 2026-08-03

**Authors:** Shasha Lan, Pei Xiang, Cui Yan, Huirong Nie, Huan Wang, Boyan Xu, Zhiyun Yang, Wen Tang, Yijuan Li, Yujian Liang, Yingqian Chen

**Affiliations:** ^1^ Department of Radiology The First Affiliated Hospital of Sun Yat‐Sen University Guangzhou China; ^2^ Medical Imaging Center The First Affiliated Hospital of Jinan University Guangzhou China; ^3^ MR Research China, GE Healthcare Beijing China; ^4^ Department of Pediatric Intensive Care Unit The First Affiliated Hospital of Sun Yat‐Sen University Guangzhou China

**Keywords:** cerebrospinal fluid, diffusion tensor imaging, gBOLD‐CSF coupling, glymphatic system, perivascular space, SMA

## Abstract

**Background:**

Spinal muscular atrophy (SMA) not only causes lower motor neuron degeneration but also affects brain development. Given the role of the glymphatic system in maintaining brain homeostasis during development, this study explores glymphatic changes in children with type 2 and 3 SMA.

**Methods:**

Forty‐eight children with SMA and 53 age‐ and sex‐matched typically developing (TD) controls were prospectively recruited. A subgroup of 22 patients received nusinersen for one year. Glymphatic function was measured using diffusion tensor image analysis along the perivascular space (DTI‐ALPS) index, frequency of enlarged perivascular space (ePVS), and the coupling strength of global blood‐oxygen‐level‐dependent (gBOLD) signals and cerebrospinal fluid (CSF) dynamics. Based on the data distribution, these parameters were compared using a two‐sample *t*‐test or Mann–Whitney U test, followed by False Discovery Rate (FDR) correction. Partial correlation analysis examined the associations between these parameters and clinical severity.

**Results:**

Compared to TD children, SMA children exhibited increased CSF volume, higher ePVS frequency, and a lower ALPS index. CSF volume was negatively correlated with SMA severity. Post‐treatment SMA patients showed increased CSF and a lower ALPS index.

**Conclusions:**

This study revealed glymphatic dysfunction in SMA and its correlation with clinical severity, highlighting a previously unrecognized feature of the disease.

## Graphical abstract

This study reveals glymphatic dysfunction in children with spinal muscular atrophy (SMA), characterized by increased cerebrospinal fluid volume and reduced ALPS index. These alterations correlate with clinical severity, identifying glymphatic dysfunction as a previously unrecognized feature of SMA pathophysiology.

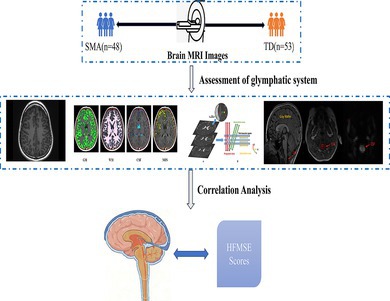

**Abbreviations**
CNS: Central nervous system
CSF: Cerebrospinal fluid
DTI‐ALPS: Diffusion tensor image analysis along the perivascular space
Dxassoc: Diffusivity along the x‐axis in association fiber area
Dxproj: Diffusivity along the x‐axis in projection fiber area
Dxsubc: Diffusivity along the x‐axis in subcortical fiber area
Dyassoc: Diffusivity along the y‐axis in association fiber area
Dyproj: Diffusivity along the y‐axis in projection fiber area
Dysubc: Diffusivity along the y‐axis in subcortical fiber area
Dzassoc: Diffusivity along the z‐axis in association fiber area
Dzproj: Diffusivity along the z‐axis in projection fiber area
Dzsubc: Diffusivity along the z‐axis in subcortical fiber area
ePVS: enlarged perivascular space
gBOLD–CSF coupling: Global blood‐oxygen‐level‐dependent signals and cerebrospinal fluid inflow dynamics
GM: Gray matter
ISF: Interstitial fluid
SMA: Spinal muscular atrophy
TD: Typically developing
VRS: Virchow‐Robin spaces
WM: White matter

## 1 Introduction

Spinal muscular atrophy (SMA) is a neurodegenerative disorder caused by mutations in the survival motor neuron 1 (SMN1) gene on chromosome 5, and its severity is primarily determined by the SMN2 copy number [1]. The incidence of SMA is approximately 1 per 10,000 live births and is second only to cystic fibrosis, a common autosomal recessive disorder [1]. The mutations of SMN1 lead to reduced expression of survival motor neuron protein. This deficiency causes the irreversible degeneration of alpha motor neurons in the spinal cord and brainstem, resulting in progressive muscle wasting and weakness. Although the emergence of various medications has improved the prognosis and extended the survival of patients with SMA, they do not fully halt motor neuron degeneration or constitute a definitive cure. Consequently, further investigation into its potential pathogenesis is essential to address unmet clinical needs and develop next‐generation treatments.

Although SMA was previously thought to involve only peripheral lower motor neurons, SMN proteins are distributed throughout the body, with the highest concentrations in the brain and the spinal cord. An increasing number of studies have indicated that SMN proteins play critical roles in brain development. Given that normal brain development depends critically on a stable internal microenvironment, and that the glymphatic system has emerged as the principal mechanism for clearing metabolic waste and preserving central nervous system (CNS) homeostasis, its functional integrity is fundamental to healthy brain development [2]. Recently, glymphatic alterations have been increasingly implicated in both neurodegenerative and neurodevelopmental disorders [3, 4, 5]. Therefore, we hypothesize that impairment of the glymphatic system may also contribute to the pathophysiology of SMA.

To date, glymphatic function has not been investigated in patients with SMA, representing a significant knowledge gap. The intrathecal injection of GBCA (gadolinium‐based contrast agents) is regarded as the current gold standard for evaluating glymphatic function. However, due to its invasive nature, its application is greatly limited in clinical patients. Fortunately, recent advances in magnetic resonance imaging (MRI) techniques offer promising, non‐invasive alternatives for evaluating both the structure and function of the glymphatic system.

The glymphatic system is a functional waste clearance network that encompasses the perivascular spaces (PVS, also known as Virchow–Robin spaces), interstitial space, arachnoid, and meningeal lymphatic vessels [6]. The PVS serve as essential channels that facilitate the exchange of cerebrospinal fluid (CSF) and interstitial fluid (ISF), a process driven primarily by arterial pulsations and respiratory pressure gradients [7]. Consequently, structural alterations in the PVS and increased intracranial CSF volume are recognized as indirect biomarkers of glymphatic dysfunction. In fact, enlarged PVS (ePVS) and increased intracranial CSF volume are frequently observed in several neurodegenerative diseases. These structural changes are considered imaging biomarkers of glymphatic dysfunction and may be precipitated by the obstruction of perivascular pathways by aggregated metabolic wastes.

A non‐invasive MRI technique, diffusion tensor image analysis along the perivascular space (DTI‐ALPS) [8], enables the assessment of water diffusion along perivascular pathways, which is a key component of glymphatic function. Although this method has some limitations, it is widely used to indirectly reflect glymphatic system function [3, 4, 9, 10]. Moreover, the coupling strength of global blood‐oxygen‐level‐dependent (gBOLD) signals and CSF inflow dynamics has also been used as a noninvasive neuroimaging indicator to evaluate glymphatic function in many studies [11, 12]. This metric reflects the essential neuro‐fluidic coupling mechanism, offering the potential to assess glymphatic efficiency and monitor therapeutic interventions.

Choosing the appropriate subject is also a critical consideration. SMA is classified into five types (types 0–IV) according to the SMN2 copy number [1]. Patients with SMA type 0‐I may have ischemic–hypoxic encephalopathy affecting the glymphatic system [1]. Adult‐onset SMA type IV presents with milder symptoms than the other types, which have very little effect on brain development [1]. Therefore, SMA types II and III represent the most appropriate population for investigating glymphatic alterations resulting from SMN protein deficiency. Accordingly, this study focused exclusively on children with SMA types II and III.

This study employed multimodal neuroimaging to evaluate the structure and function of the glymphatic system in pediatric patients with SMA types II and III. The aims of this study were to (a) determine whether type 2 and 3 SMA patients exhibit abnormal PVS and CSF accumulation compared to healthy controls, (b) investigate whether patients with type 2 and 3 SMA have impaired glymphatic function, (c) assess the association between glymphatic parameters and clinical severity, and (d) evaluate glymphatic changes following one year of nusinersen treatment.

## 2 Materials and Methods

### 2.1 Participants

The study protocol (No. [2021]710) was approved by the Institutional Review Board of the First Affiliated Hospital, Sun Yat‐sen University. Informed consent was obtained from the guardians of all participants according to the ethical principles outlined in the Declaration of Helsinki.

Children with SMA were recruited from the First Affiliated Hospital of Sun Yat‐sen University between January 2022 and November 2023. The inclusion criteria were as follows: (1) right‐handedness; (2) age ranging from 5 to 18 years; (3) type 2 and 3 SMA; (4) treatment‐naïve. The exclusion criteria were the following: (1) refusal to participate; (2) contraindications to MRI; (3) previously treated with any specific medicine for SMA; (4) history of brain injury or other psychiatric/neurological disorders; and (5) poor image quality due to scanning or motion artifacts, as determined by two experienced neuroradiologists. Finally, 48 SMA patients were included in this study. In addition, 53 age‐ and sex‐matched typically developing (TD) individuals were enrolled from the community and subjected to the same exclusion criteria as the SMA patients. Twenty‐two of these patients with SMA were treated with a one‐year standard course of intrathecal nusinersen.

### 2.2 Clinical Assessment

Genetic testing was conducted on every individual with SMA to confirm the diagnosis of SMN1‐related SMA. Current consensus criteria were utilized for classification [1]. Global motor impairment in SMA patients was measured using the Hammersmith Functional Motor Scale‐Expanded (HFMSE), which evaluates daily activities across 33 categories of motor function. The scores range from 0 to 66, with higher scores indicating superior motor function.

### 2.3 MRI Data Acquisition

Images of all subjects were obtained using a 3.0 T MRI scanner (SIGNA Pioneer, GE Healthcare, Waukesha, WI, USA) with a 32‐channel head coil. During the scanning procedure, all participants were instructed to remain still and supine. After localizers, the coronal T2‐weighted sequence was obtained to exclude any cranial lesion [13]. Structural images of the whole brain were obtained using a three‐dimensional (3D) fast spoiled gradient‐echo sequence: Repetition time (TR) = 7.5 ms, echo time (TE) = 3.1 ms, matrix = 256 × 256, slice thickness = 1 mm with no slice gap, field of view (FOV) = 256 mm × 256 mm, and a total of 188 slices. For diffusion images, we used a single‐shot EPI sequence: TR = 10,000 ms, TE = 88.6 ms, FOV = 224 mm × 224 mm, matrix = 112 × 112, b = 1000 s/mm^2^, slice thickness = 2 mm, no slice gap, voxel size = 2 mm × 2 mm × 2 mm, NEX = 1; 32 gradient directions, and a total of 75 slices. The synthetic MRI (SyMRI) scan is a two‐dimensional multiple‐dynamic multiple‐echo (MDME) pulse sequence and comprises four automatically calculated saturation delay times and two echo times. The parameters of SyMRI scan were as follows: TR = 10205.0 ms, TE = 10.9 ms, filp angle = 20°, thickness = 2 mm/no gap, NEX = 1.00, ETL = 16, pixel size = 2.0 mm × 2.0 mm, scanning time = 5.5 min, and a total of 225 slices. Resting‐state functional MRI was performed using an echo‐planar image sequence: TR = 2,000 ms, TE = 30 ms, flip angle = 90°, FOV = 224 mm × 224 mm, slice thickness = 3.5 mm, voxel size = 3.5 mm × 3.5 mm × 3.5 mm, 200 time points, and a total of 7200 slices.

### 2.4 Identification of Enlarged PVS

The severity of ePVS was independently assessed on axial T1‐weighted images by two neuroradiologists with more than 5 years of work experience using axial T1‐weighted images using a validated 5‐point visual rating scale (detailed in Supporting Information). The concordance between the two raters was determined using Kendall's W [14, 15, 16].

### 2.5 Calculation of CSF Volume and Other Brain Component Volume

The R1, R2, and proton density (PD) maps were first estimated from the raw MDME data using SyMRI software (version 8.0.4, Synthetic MR, Linköping, Sweden). These values can be used to segment the brain tissue into white matter (WM), gray matter (GM), and CSF. Brain parenchymal volume (BPV) is the sum of WM, GM, and non‐WM/GM/CSF (NoN), whereas intracranial volume (ICV) is the sum of BPV and CSF. We obtained the volumes of the WM, GM, CSF, NoN, BPV, and ICV using a specific post‐processing software (SyMRI), which can automatically segment and quantify the brain tissues using magic images [17].

### 2.6 Calculation of the Diffusivities and ALPS Index

The PANDA toolbox (State Key Laboratory of Cognitive Neuroscience and Learning, Beijing Normal University) was used to calculate the diffusion metric images [18]. After standard preprocessing for head motion and eddy current distortions [8], diffusivities along the x‐ (Dx), y‐ (Dy), and z‐axes (Dz) were measured in the projection, association, and subcortical fiber areas [5]. Two neuroradiologists independently placed spherical regions of interest (ROIs, 5 mm diameter) in these regions on a slice of the lateral ventricle body. Average values were used for the analysis. All subjects were right‐handed; therefore, the ROIs were placed in the left hemisphere [5]. To minimize the effect of the periventricular white matter hyperintensities (PVH), the ALPS index was calculated using the following formula: ALPS index = mean (Dxproj, Dxassoc)/mean (Dyproj, Dzassoc) [10].

### 2.7 Coupling Between BOLD Signal and CSF Flow

To assess gBOLD‐CSF coupling, resting‐state functional MRI images were preprocessed using the 1000 functional connectomes project script (version 1.1‐beta) [12]. The detailed steps are provided in the Supporting Information.

### 2.8 Statistical Analysis

Statistical analyses were performed using SPSS version 25.0. Normality of data distribution was assessed using the Shapiro–Wilk test. Continuous variables are presented as means and standard deviations (SD), and categorical variables are presented as frequencies and proportions. For comparisons between the SMA and TD groups, the two‐sample *t*‐test or Mann–Whitney U test was used for continuous variables, as appropriate, and the χ [2] test for categorical variables. Group differences in ePVS severity were assessed using the Mann–Whitney U test. To control for multiple comparisons, the Benjamini–Hochberg false discovery rate (FDR) procedure was applied to the diffusivity analyses. Inter‐observer agreement on the ePVS assessment between the two experts was evaluated using Kendall's W. Partial correlation analysis, with age and sex as covariates, was used to assess the relationship between imaging parameters and clinical severity. For the treatment subgroup, changes in quantitative parameters were assessed using paired *t*‐tests, and *p*‐values were adjusted using the Benjamini–Hochberg FDR procedure to control for multiple comparisons.

## 3 Results

### 3.1 Clinical Characteristics

A total of 48 children with SMA (age range, 5–18 years; mean age = 9.65 ± 4.10 years; 28 males and 20 females) and 53 TD children (age range, 5–18 years; mean age = 9.79 ± 3.21 years; 37 males and 16 females) were included in this study. The demographic and clinical characteristics of all participants are summarized in Table 1.

**TABLE 1:** Demographic and clinical characteristics of the SMA and TD groups.

Characteristics	TD (*n* = 53)	SMA (*n* = 48)	*p*
Age (mean ± SD)	9.79 ± 3.21	9.65 ± 4.10	0.841
Age range (years)	5–18	5–18	—
Gender (M/F)	37/16	28/20	0.23
SMA type (2/3)	—	31/17	—
HFMSE (mean ± SD)	—	24.5 ± 18.7	—
Range	—	0–65	—

Abbreviations: F, Female; HFMSE, Hammersmith Functional Motor Scale–Expanded; M, Male; SD, Standard deviation; SMA, Spinal Muscular Atrophy; TD, Typically developing.

### 3.2 Comparison of Frequency of ePVS Between SMA and TD Children

**FIGURE 1:**
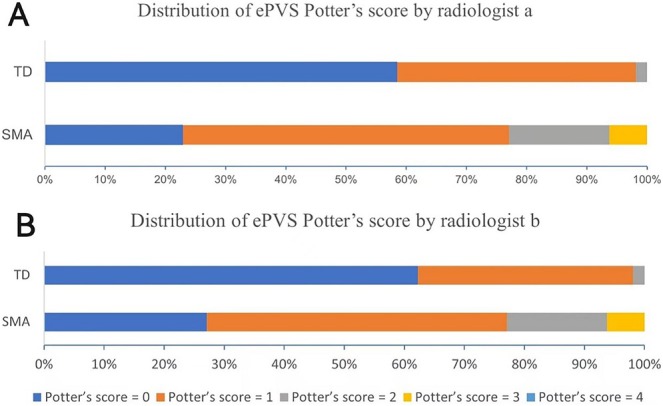
Potter's score distribution. The bar plot represents the distribution of Potter's score for ePVS by Radiologist A (A) and Radiologist B (B) in typically developing (TD) and SMA patients; bar width reflects the number of subjects in each group. ePVS, enlarged perivascular space.

The results of the ePVS assessment are presented in Table 2 and Figure 1. Kendall's W coefficient for inter‐rater agreement was 0.944, indicating excellent concordance between the two raters. The frequency of ePVS was higher in the SMA group than in the controls (*p* < 0.001).

**TABLE 2:** The level of ePVS in SMA and TD children.

ePVS level	TD children		SMA children	
	Radiologist A	Radiologist B	Radiologist A	Radiologist B
0	31 (58.5%)	33 (62.3%)	11 (22.9%)	13 (27.1%)
1	21 (39.6%)	19 (35.8%)	26 (54.2%)	24 (50.0%)
2	1 (1.9%)	1 (1.9%)	8 (16.7%)	8 (16.7%)
3	0 (0%)	0 (0%)	3 (6.2%)	3 (6.2%)
4	0 (0%)	0 (0%)	0 (0%)	0 (0%)

*Note: p*‐value less than 0.05 indicates statistical significance. This table presents the visual assessment of ePVS burden on brain MRI scans, scored by two independent radiologists (Radiologist A and Radiologist B) using a validated 5‐point semi‐quantitative scale (0–4), where 0 = none/minimal, 1 = mild, 2 = moderate, 3 = severe, and 4 = most severe. For each diagnostic group (TD and SMA), the number of children assigned to each ePVS severity level is shown as absolute counts with the corresponding percentage (in parentheses) of the group total. Percentages are calculated separately for each rater's assessment.

Abbreviations: ePVS, enlarged perivascular spac; SMA, Spinal Muscular Atrophy; TD, Typically developing.

### 3.3 SyMRI Findings

**FIGURE 2:**
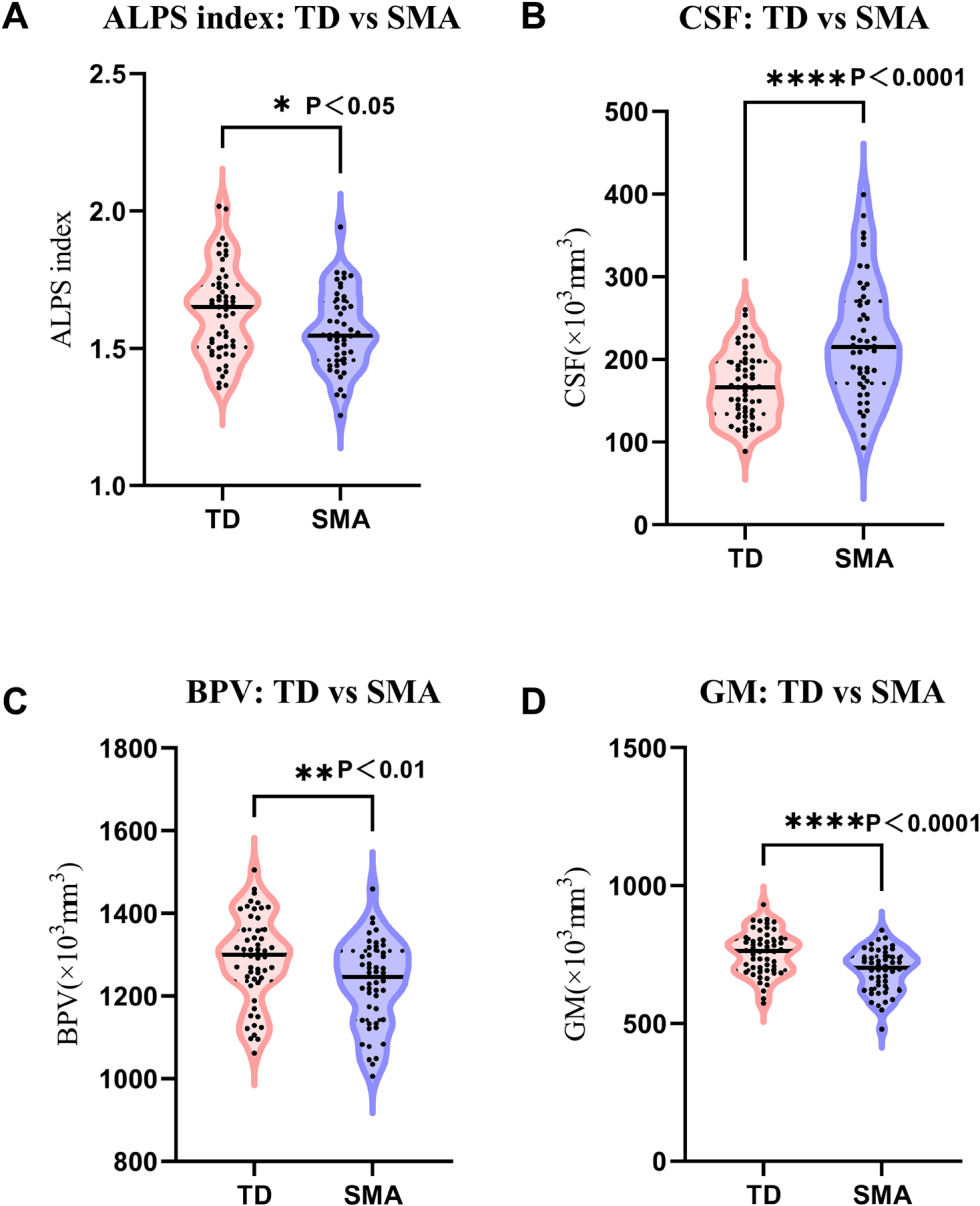
Comparison of the brain volumetric parameters and the ALPS index between SMA and TD groups. SMA, Spinal Muscular Atrophy; TD, Typically developing; CSF, Cerebrospinal fluid; BPV, parenchymal volume; GMV, gray matter volume; *p*‐value less than 0.05 indicates statistical significance.

The results of brain segmentation are shown in Table 3 and Figure 2. Statistical analysis demonstrated that the ICV did not show any statistical difference between the SMA and control groups. The WM volume, GM volume, BPV, WMV/ICV, and GMV/ICV were significantly smaller in the SMA group than in the control group. In addition, the CSF, NoN, and NON/ICV ratios were higher in patients with SMA.

**TABLE 3:** Comparison of brain volumetric parameters between SMA and TD children.

	TD (M ± SD)	SMA (M ± SD)	Adjusted *p*
WMV (x10 [3] mm^3^)	419.09 ± 53.06	389.73 ± 47.73	**0.005**
GMV (x10^3^mm^3^)	755.03 ± 78.90	691.73 ± 78.00	**< 0.001**
CSF (x10^3^mm^3^)	167.87 ± 40.99	221.91 ± 71.40	**< 0.001**
NON (x10^3^mm^3^)	115.13 ± 42.59	146.12 ± 57.07	**0.003**
BPV (x10^3^mm^3^)	1289.34 ± 106.15	1227.56 ± 102.73	**0.005**
ICV (x10^3^mm^3^)	1456.15 ± 116.48	1449.42 ± 133.24	0.787
WMV/ICV	0.288 ± 0.030	0.269 ± 0.027	**0.002**
GMV/ICV	0.518 ± 0.034	0.478 ± 0.043	**< 0.001**
NoN/ICV	0.079 ± 0.028	0.101 ± 0.039	**0.002**

*Note:* Data are presented as the M ± SD (M mean, SD standard deviation). False discovery rate‐adjusted *p*‐values less than 0.05 indicate statistical significance. Bold values indicate statistically significant differences (adjusted *p* < 0.05) between the SMA and TD groups.

Abbreviations: SMA, Spinal Muscular Atrophy; TD, typically developing; WMV, white matter volume; GMV, gray matter volume; CSF, cerebrospinal fluid; NON, non‐WM/GM/CSF; BPV, parenchymal volume; ICV, intracranial volume.

### 3.4 Comparison of Diffusivities and the ALPS Index Between SMA and TD Children

The results of the diffusivities and the ALPS index are shown in Table 4 and Figure 2. The ALPS index was significantly lower in the SMA group than in the TD group (*p* < 0.05).

**TABLE 4:** Comparison of the diffusivities and ALPS index between SMA and TD groups.

Diffusivity	TD (M ± SD)	SMA (M ± SD)	Adjusted *p*
Dxproj (×10^−3^ mm^2^/s)	0.652 ± 0.060	0.660 ± 0.079	0.564
Dxassoc (×10^−3^ mm^2^/s)	0.840 ± 0.121	0.744 ± 0.097	**< 0.001**
Dxsubc (×10^−3^ mm^2^/s)	0.929 ± 0.091	0.951 ± 0.112	0.308
Dyproj (×10^−3^ mm^2^/s)	0.426 ± 0.053	0.605 ± 0.204	**< 0.001**
Dyassoc (×10^−3^ mm^2^/s)	1.068 ± 0.136	1.123 ± 0.121	**0.047**
Dysubc (×10^−3^ mm^2^/s)	0.814 ± 0.134	0.711 ± 0.187	**0.005**
Dzproj (×10^−3^ mm^2^/s)	1.197 ± 0.069	1.061 ± 0.219	**< 0.001**
Dzassoc (×10^−3^ mm^2^/s)	0.487 ± 0.074	0.461 ± 0.084	0.136
Dzsubc (×10^−3^ mm^2^/s)	0.734 ± 0.121	0.819 ± 0.172	**0.008**
ALPS index	1.64 ± 0.17	1.56 ± 0.14	**0.028**

*Note:* Data are presented as the M ± SD (M mean, SD standard deviation). False discovery rate‐adjusted *p*‐values less than 0.05 indicate statistical significance. Bold values indicate statistically significant differences (adjusted *p* < 0.05) between the SMA and TD groups.

Abbreviations: SMA, Spinal Muscular Atrophy; Dxproj, diffusivity along the x‐axis in projection fiber area; Dxassoc, diffusivity along the x‐axis in association fiber area; Dxsubc, diffusivity along the x‐axis in subcortical fiber area; Dyproj, diffusivity along the y‐axis in projection fiber area; Dyassoc, diffusivity along the y‐axis in association fiber area; Dysubc, diffusivity along the y‐axis in subcortical fiber area; Dzproj, diffusivity along the z‐axis in projection fiber area; Dzassoc, diffusivity along the z‐axis in association fiber area; Dzsubc, diffusivity along the z‐axis in subcortical fiber area.

### 3.5 Comparison of gBOLD‐CSF Coupling Between SMA and TD Children

In this study, gBOLD‐CSF coupling strengths after excluding head motion were calculated for twenty‐five children with SMA and 21 children with TD. We found a significant cross‐correlation between the gBOLD and CSF signals in all subjects (*p* < 0.05). The mean gBOLD‐CSF cross‐correlation function displayed a positive peak (mean *r* = 0.168, all *p* < 0.001) at time lags of –4 s and a negative peak (mean r = −0.143, all *p* < 0.001) at time lags of +6 s. At the same time, the negative first‐order derivative of the gBOLD signal was correlated to the CSF signal (*p* < 0.001; mean d/dt r = −0.193, group mean lag = 0 s). The results are shown in Figure S2 in the Supporting Information. Although the strength of gBOLD‐CSF coupling tended to decline in the SMA group compared to the TD group (r: 0.205 ± 0.144 vs. 0.244 ± 0.161), there was no statistically significant difference in the strength of gBOLD‐CSF coupling between the two groups.

### 3.6 Comparison of Parameters Related to Glymphatic System Before and After One Year's Treatment With Nusinersen

There was no statistically significant difference in the frequency of ePVS between the posttreatment group and pretreatment (details in Table S1 and Figure S1 of the Supporting Information).

### 3.7 Comparison of Brain Volumetric Parameters Between Children With SMA Pretreatment and Posttreatment

The brain volumetric parameters are presented in Table S2 (Supporting Information). Posttreatment SMA children showed significantly larger CSF volume compared to baseline.

### 3.8 Comparison of Diffusivities and the ALPS Index Between Children With SMA Pretreatment and Posttreatment

The results of the diffusivities and the ALPS index are shown in Table S3 in the Supporting Information. Dzassoc was higher in children with SMA posttreatment. The ALPS index, Dyproj, and Dyassoc were significantly lower in the posttreatment than in the pretreatment SMA children (*p* < 0.05). In addition, no significant differences were found in Dxproj, Dxassoc, Dxsubc, Dysubc, Dzproj, or Dzsubc between the two groups (*p* > 0.05).

### 3.9 Partial Correlation Analyses

The CSF volume was negatively correlated with the HFMSE scores (r = −0.356, *p* < 0.05), whereas no correlation with statistical significance was found between the ALPS index and the HFMSE scores (r = −0.341, *p* = 0.065).

## 4 Discussion

This multimodal MRI study provides initial evidence of possible glymphatic system dysfunction in children with type 2 and 3 SMA. Compared to TD children, children with type 2 and 3 SMA exhibited significant CSF accumulation, enlarged perivascular spaces (ePVS), and a reduced DTI‐ALPS index, suggesting impaired CSF drainage and altered glymphatic function. Moreover, CSF volume was negatively correlated with HFMSE scores, suggesting an association between glymphatic dysfunction and clinical severity. Furthermore, in the subset of patients who underwent a follow‐up scan after nusinersen treatment, we observed a trend toward a further decrease in the ALPS index and an increase in CSF volume. These changes are not consistent with the expectation that treatment would improve glymphatic function. Rather, they indicate a more complex acute physiological response, underscoring the need for further mechanistic investigations.

### 4.1 MRI Parameters and the Glymphatic System

The glymphatic system is a macroscopic waste clearance pathway in the CNS that depends on a network of perivascular channels. It functions by driving convective fluid flow within these PVS, which serve as essential conduits around cerebral vessels. As the only component of this system directly visible on MRI, the PVS serve as a key in vivo biomarker for assessing glymphatic anatomy [19]. Therefore, alterations in PVS morphology, such as the volume increases observed following impaired CSF flow, provide critical insights into the dysfunctional state of the glymphatic system [8]. In this study, an increased PVS burden was observed in children with SMA, indicating that CSF drainage may be obstructed.

The PVS extend along the penetrating cerebral vessels and provides the primary conduit for convective glymphatic flow. The DTI‐ALPS method quantifies this process by comparing water diffusivity along the perivascular direction (parallel to the projection fibers) to that perpendicular to this direction (parallel to the association fibers). Theoretically, a higher ratio suggests higher water diffusivity along the PVS, which is indicative of active glymphatic flow. Conversely, a lower ratio implies impaired perivascular diffusivity [5]. The validity of this method has been widely demonstrated in neurodegenerative and neurodevelopmental disorders [4, 5, 8]. In this study, the ALPS index was nearly 5% lower in children with SMA compared to TD controls, suggesting a potential reduction in glymphatic clearance activity in SMA, though this indirect marker requires further validation.

Furthermore, the physiological mechanism underlying gBOLD–CSF coupling can be summarized as follows: During NREM sleep, slow neuronal oscillations drive rhythmic hemodynamic fluctuations, which reflect periodic changes in cerebral blood volume. Because intracranial volume is fixed, a decrease in blood volume creates space that is immediately filled by inflowing CSF, manifesting as a bright signal in the fourth ventricle (inflow enhancement). EEG slow waves precede BOLD changes, which in turn precede CSF signals by approximately 6.4 s. Hence, the strength of gBOLD–CSF coupling serves as an indirect marker of neurovascular–glymphatic coordination [20]. While the gBOLD–CSF coupling strength showed a trend toward reduction in the SMA group compared to the TD group, this difference was not statistically significant. The lack of significance may be attributable to the limited statistical power, as the final sample size was reduced after excluding participants with excessive head motion.

Additionally, we found a correlation between glymphatic dysfunction and SMA severity, the causal direction of which remains to be determined. Given the observational nature of this study, these findings should be considered hypothesis‐generating. Based on our findings, PVS burden, ALPS index, and CSF volume, each of which reflects a distinct aspect of glymphatic dysfunction and fluid homeostasis, may serve as candidate biomarkers. Future studies should specifically investigate their ability to monitor disease progression and treatment effects in pediatric patients with SMA.

### 4.2 Potential Mechanisms of Glymphatic System Dysfunction in SMA

The glymphatic system is conceptualized as a convective clearance circuit within the CNS, whose effective functioning depends on precise coordination between CSF dynamics, perivascular flow, and astrocytic endfoot function, with particular emphasis on the polarized distribution of AQP4 [10].

SMA is a neurodegenerative disease caused by biallelic mutations in the SMN1 gene and a consequent systemic deficiency of SMN protein [21]. The SMN protein has a widespread impact on developmental processes of the CNS [22]. Our previous studies have also indicated that children with type 2 and 3 SMA show widespread structural changes in both brain gray matter and white matter, as well as altered brain connectivity [23, 24]. Our study revealed glymphatic dysfunction in SMA, providing a novel perspective on the pathology of this disease. In neurodegenerative diseases such as Alzheimer's and Parkinson's diseases, reactive astrogliosis is frequently accompanied by a loss of AQP4 polarization [25]. Depolarization of AQP4 has been conclusively established as a central mechanism underlying the impairment of glymphatic clearance function in these disorders [26]. McGivern et al. have demonstrated that astrocytes in SMA exhibit early reactive activation characterized by GFAP upregulation and process retraction, as well as functional impairments including elevated basal calcium levels and diminished calcium response to ATP [27]. Therefore, it is plausible that a similar disruption of AQP4 polarization occurs in SMA astrocytes, which may drive glymphatic dysfunction and exacerbate disease progression by impairing the clearance of toxic metabolites from the motor neuron microenvironment. However, whether AQP4 polarization is disrupted in SMA and whether it serves as a direct link between astrocytic pathology and glymphatic dysfunction require rigorous experimental validation in future studies.

Furthermore, activated astrocytes secrete pro‐inflammatory mediators [27, 28], which perpetuate the cycle of glial activation, leading to further morphological alterations in astrocytes and microglia [29]. Previous studies have revealed elevated levels of various pro‐inflammatory cytokines and neurotrophic factors in the CSF of patients with SMA, indicating the presence of a significant pro‐inflammatory milieu in the CNS [28]. This inflammatory milieu also promotes the recruitment and perivascular accumulation of infiltrating immune cells. The combined effect of these changes may synergistically compromise the glymphatic conduit, resulting in significantly reduced brain clearance capacity [30]. However, a critical unanswered question is whether glymphatic dysfunction is a secondary outcome of these broad structural changes or a primary contributor. Resolving this issue will require longitudinal studies using preclinical models to determine the temporal sequence of events and experimental manipulations specifically targeting the glymphatic system.

Therapeutic strategies to restore the SMN protein are now well‐established, with the antisense oligonucleotide nusinersen, the gene therapy onasemnogene abeparvovec, and the oral splicing modifier risdiplam widely used in clinical practice [31]. Consistent with established efficacy, patients in our cohort who received one year of nusinersen treatment demonstrated a trend toward clinical improvement. However, the impact of intrathecal administration on the CNS microenvironment, specifically on CSF dynamics and waste clearance, remains a critical scientific question. Several studies have reported that nusinersen treatment induces alterations in CSF, including fluctuations in the levels of neurofilament light chain (NfL), β‐amyloid (Aβ), and total protein [32, 33]. These changes may be related to disease activity, neuronal repair, or altered clearance dynamics. Previous studies have established a close association between the accumulation of Aβ protein in the CSF and impaired glymphatic system function in Alzheimer's disease [34]. Similarly, alterations in Aβ metabolism have been implicated in SMA, with studies reporting significant changes in CSF Aβ levels following disease‐modifying treatment [35]. Based on this, one might intuitively predict that enhanced neuronal repair following nusinersen treatment would lead to increased Aβ clearance and decreased CSF Aβ levels. However, several studies have reported a paradoxical rise in CSF Aβ after treatment, possibly due to enhanced neuronal metabolic activity and increased Aβ production [35]. In our study, we observed increased CSF volume and decreased ALPS index post‐treatment, and these changes may share a common mechanism with Aβ elevation. These counterintuitive findings challenge prevailing assumptions about post‐treatment biomarker trajectories and necessitate further mechanistic investigations to elucidate the underlying interactions between neuronal metabolism, protein clearance, and CSF dynamics in the SMA.

## 5 Limitations

This study has several limitations. First, the cross‐sectional design of the main cohort precludes causal inference. The observed correlations do not establish whether glymphatic dysfunction drives disease progression or is a consequence of neurodegeneration. Second, the methodological limitations of the DTI‐ALPS index must be considered, as its specificity for perivascular diffusivity may be confounded by the adjacent white matter anatomy and its ROI placement introduces operator‐dependent variability. Therefore, future work must address these specific issues to improve the validity of the metric by developing biophysical models to correct for structural confounders and establishing automated, standardized pipelines for ROI definition to enhance reproducibility. Third, due to the lack of T2‐weighted images, the ePVS severity on T1‐weighted images was manually assessed. As this method can be labor‐intensive and subject to variability, future studies will incorporate coregistered T1‐ and T2‐weighted sequences to perform automated quantification of PVS volume [12]. Fourth, a particularly critical methodological limitation arises from the treatment modality. All patients received nusinersen via repeated intrathecal lumbar punctures. This procedure is known to transiently alter CSF pressure and may affect intracranial fluid distribution, potentially inducing subtle, procedure‐related microstructural changes along CSF pathways. This constitutes a potential confounder in that our current observational design cannot be fully disentangled. Fifth, the present study focused exclusively on global BOLD (gBOLD)–CSF coupling. Future investigations could explore regional differences in BOLD–CSF coupling across cortical areas, particularly the motor and frontal cortices, to provide a more nuanced understanding of region‐specific glymphatic function. Finally, the statistical power of this study is limited by its small sample size. Future studies should recruit a larger cohort or adopt a multicenter design to enhance the generalizability and robustness of the findings.

## 6 Conclusions

This multimodal MRI study provides the first in vivo evidence of structural and functional impairment of the glymphatic system in children with type 2 and 3 SMA. The correlation between these imaging features and clinical severity suggests that glymphatic alterations may be associated with disease manifestations. Further studies with larger, longitudinal cohorts are needed to validate these observations and elucidate the complex relationship between SMN restoration, CSF dynamics, and glymphatic function.

## Author Contributions

Shasha Lan, Pei Xiang and Cui Yan: Conception, study design, data acquisition, statistical analysis, writing – original draft, writing – review and editing. Huirong Nie, Huan Wang, Boyan Xu: Acquisition, analysis and interpretation of data. Zhiyun Yang, Wen Tang and Yijuan Li: Conception, study design, writing – review and editing. Yujian Liang and Yingqian Chen: Conception, study design, funding acquisition, formal analysis, writing – review and editing. All authors read and approved the final manuscript.

## Funding

This research was supported by the Natural Science Foundation of Guangdong Province (Grant No. 2025A1515011545) and the Research Foundation of Sun Yat‐sen University (Grant No. 80000‐3160029).

## Ethics Statement

Institutional Review Board approval was obtained from the First Affiliated Hospital of Sun Yat‐sen University (No. [2021]710).

## Consent

Written informed consent was obtained from all subjects (patients) in this study.

## Conflicts of Interest

The authors declare no conflicts of interest.

## Supporting information


**Table S1:** The level of ePVS in the pretreatment and posttreatment SMA children.
**Table S2:** Comparison of brain volumetric parameters between pretreatment and posttreatment SMA children.
**Table S3:** Comparison of the diffusivities and ALPS index between pretreatment and posttreatment SMA children.
**Figure S1:** Potter's score distribution. The bar plot represents the distribution of Potter's score for ePVS by Radiologist A (A) and Radiologist B (B) in pretreatment and posttreatment SMA patients; bar width reflects the number of subjects in each group.
**Figure S2:** (A) The bottom slice of the axial T1W and rsfMRI images. The red area was used to extract the CSF signal. (B) The mean gBOLD‐CSF crosscorrelation function (*N* = 46) was characterized by a positive peak (r = 0.168, p < 0.001; permutation test with *n* = 10,000) around a lag of −4 s and a negative peak (r = −0.143, p < 0.001; permutation test with *n* = 10,000) at a lag of +6 s. The cross‐correlation function between the CSF signal and the negative derivative of the gBOLD signal was also calculated, characterized by a large positive peak around a lag of 0 s (d/dt r = −0.193, p < 0.001; permutation test of *n* = 10,000). Gray dashed lines denote the 95% confidence interval of the null distribution. Abbreviation: gBOLD, global blood‐oxygen‐level‐dependent signal.

## Data Availability

The data that support the findings of this study are available from the corresponding author upon reasonable request.
