# Supplementary material for: Glymphatic Dysfunction in Children With Type 2 and 3 Spinal Muscular Atrophy
Source: CNS Neurosci Ther. 2026 Aug 3;32(8):e71063. doi: 10.1002/cns.71063 (PMC13431131; doi:10.1002/cns.71063)
Supplement: Supplementary file 1 — Table S1: The level of ePVS in the pretreatment and posttreatment SMA children. Table S2: Comparison of brain volumetric parameters between pretreatment and posttreatment SMA children. Table S3: Comparison of the diffusivities and ALPS index between pretreatment and posttreatment SMA children. Figure S1: Potter's score distribution. The bar plot represents the distribution of Potter's score for ePVS by Radiologist A (A) and Radiologist B (B) in pretreatment and posttreatment SMA patients; bar width reflects the number of subjects in each group. Figure S2: (A) The bottom slice of the axial T1W and rsfMRI images. The red area was used to extract the CSF signal. (B) The mean gBOLD‐CSF crosscorrelation function (N = 46) was characterized by a positive peak (r = 0.168, p < 0.001; permutation test with n = 10,000) around a lag of −4 s and a negative peak (r = −0.143, p < 0.001; permutation test with n = 10,000) at a lag of +6 s. The cross‐correlation function between the CSF signal and the negative derivative of the gBOLD signal was also calculated, characterized by a large positive peak around a lag of 0 s (d/dt r = −0.193, p < 0.001; permutation test of n = 10,000). Gray dashed lines denote the 95% confidence interval of the null distribution. Abbreviation: gBOLD, global blood‐oxygen‐level‐dependent signal. [file CNS-32-e71063-s001.docx]

**Supplementary Materials**

This supplemental material has been provided by the authors to give readers additional information about their work

**Identification of Enlarged Perivascular Spaces (ePVS)**

Two neurologists, with over 5 years work experience, who independently assessed he level of ePVS on axial T1w images using a validated 5-point visual rating scale. The standard involves a visual assessment of T1w or T2w images, obtaining slices from the centrum semiovale, basal ganglia region. The number of PVS were manually counted after selecting the axial slice with the highest visual assessment of PVS burden. According to the number of PVS, the severity of ePVS were classified into 5 grades: 0 (none), 1 (1–10), 2 (11–20), 3 (21–40), and 4 (>40)^1^

**Coupling between BOLD Signal and CSF Flow**

Every original rsfMRI data in turn underwent removing the first 10 time points, slice-timing correction, realignment, bandpass filtering (0 Hz < f < 0.1 Hz) , linear and quadratic detrending by the Statistical Parametric Mapping 12 (SPM12) (http://www.fil.ion.ucl.ac.uk/spm/) and the Data Processing Assistant for Brain Imaging (DPABI) (http://rfmri. org/DPABI) based on MATLAB^2^. Nuisance regression analyses of gBOLD signals, CSF signals, and motor parameters were not performed because these variables were the highlights of current research^3^. The gBOLD signal was obtained by extracting and averaging in whole brain grey matter regions, the whole brain grey matter was defined according to Automated Anatomical Labeling 2(AAL2)^4^. For estimation of the regional distribution of lymphatic function, the AAL2 atlas was separated into anterior, middle, and posterior sections in accordance with the labels of frontal, temporoparietal, and occipital regions The fMRI signals within the individual's gray matter mask were extracted and averaged after all the preprocessing procedures. At the same time, the CSF was obtained from the bottom slice (around the base of the cerebellum) of the fMRI image in order to maximise the sensitivity to the CSF inflow effect. The CSF masks were manually defined on the bottom slice of the fMRI image and further located by using T1-weighted structural images^2^. Then, we extracted the average time series of CSFs according to the signal strength of each subject. The cross-correlation function between the gBOLD and CSF signals was calculated in order to quantify the gBOLD-CSF coupling at various time lags (-20 to 20s)^2^. The correlation coefficients from the negative peaks at lags +6 s were used to quantify the strength of gBOLD-CSF coupling. Since the positive peak at -4s has the same amplitude as the negative peak at +6s, the BOLD–CSF correlation at this negative peak was used to quantify the strength of BOLD–CSF coupling. Besides, the cross-correlation function between the negative derivative of the gBOLD signal and the CSF signal was calculated. We randomly matched the gBOLD time series and CSF time series from various sujects and calculated the coupling index to validate the the statistical significance of the gBOLD-CSF coupling, and repeated this procedure 10,000 times to acquire the a null distribution^2^.

**References**

1. Potter GM, Chappell FM, Morris Z, Wardlaw JM. Cerebral perivascular spaces visible on magnetic resonance imaging: development of a qualitative rating scale and its observer reliability. *Cerebrovasc Dis*. 2015;39(3-4):224-231. doi:10.1159/000375153

2. Zhang Y, Peng B, Chen S, et al. Reduced coupling between global signal and cerebrospinal fluid inflow in patients with depressive disorder: A resting state functional MRI study. *J Affect Disord*. 2024;354:136-142. doi:10.1016/j.jad.2024.03.023

3. Fultz NE, Bonmassar G, Setsompop K, et al. Coupled electrophysiological, hemodynamic, and cerebrospinal fluid oscillations in human sleep. *Science*. 2019;366(6465):628-631. doi:10.1126/science.aax5440

4. Jiang D, Liu L, Kong Y, et al. Regional Glymphatic Abnormality in Behavioral Variant Frontotemporal Dementia. *Ann Neurol*. 2023;94(3):442-456. doi:10.1002/ana.26710

**Table S1. The level of ePVS in the pretreatment and posttreatment SMA children**

| ePVS level | Pretreatment SMA | | Posttreatment SMA | |
| --- | --- | --- | --- | --- |
|  | radiologist a radiologist b | | radiologist a radiologist b | |
| 0 | 5(22.7%) | 5(22.7%) | 6(27.3%) | 7(31.8%) |
| 1 | 13(59.1%) | 13(59.1%) | 14 (63.6%) | 13(59.1%) |
| 2 | 3(13.6%) | 3(13.6%) | 2(9.1%) | 2(9.1%) |
| 3 | 1(4.6%) | 1(4.6%) | 0(0%) | 0(0%) |
| 4 | 0(0%) | 0(0%) | 0(0%) | 0(0%) |

SMA, Spinal Muscular Atrophy; TD, Typically developing; ePVS ; enlarged perivascular space

This table presents the visual assessment of ePVS burden on brain MRI scans, scored by two independent radiologists (Radiologist a and Radiologist b) using a validated 5‑point semi‑quantitative scale (0–4), where 0 = none/minimal, 1 = mild, 2 = moderate, 3 = severe, and 4 = most severe. For each diagnostic group (TD and SMA), the number of children assigned to each ePVS severity level is shown as absolute counts with the corresponding percentage (in parentheses) of the group total. Percentages are calculated separately for each rater’s assessment.

p value less than 0.05 indicate statistical significance.

**Table S2. Comparison of brain volumetric parameters between** **pretreatment and posttreatment SMA children**

|  | Pretreatment SMA(M±SD) | Postreatment SMA (M±SD) | adjusted *p*-value |
| --- | --- | --- | --- |
| WMV(x10^3^mm^3^) | 388.22±32.15 | 402.62±50.45 | 0.416 |
| GMV(x10^3^mm^3^) | 693.62±71.25 | 672.25±81.70 | 0.0.416 |
| CSF(x10^3^mm^3^) | 204.80±59.21 | 222.83±66.92 | ***0.039*** |
| NON(x10^3^mm^3^) | 143.84±59.16 | 150.63±62.91 | 0.807 |
| BPV(x10^3^mm^3^) | 1225.67±99.13 | 1225.52±102.52 | 0.987 |
| ICV(x10^3^mm^3^) | 1430.38±117.09 | 1448.29±124.18 | *0.176* |
| WMV/ICV | 0.273±0.025 | 0.278±0.029 | 0.696 |
| GMV/ICV | 0.485±0.038 | 0.465±0.050 | 0.204 |
| NON/ICV | 0.099±0.038 | 0.104±0.041 | 0.807 |

Data is presented as the mean±SD (*p* value < 0.05). SMA, Spinal Muscular Atrophy; TD, typically developing; WMV, white matter volume; GMV, gray matter volume; CSF, cerebrospinal fluid; NON, non-WM/GM/CSF; BPV, parenchymal volume; IVC, intracranial volume

**False discovery rate-adjusted p-values less than 0.05 indicate statistical significance*.

**Table S3. Comparison of the diffusivities and ALPS index between pretreatment and posttreatment SMA children**

| Diffusivity | Pretreatment SMA(M±SD) | Postreatment SMA (M±SD) | adjusted *p*-value |
| --- | --- | --- | --- |
| Dxproj(×10^-3^mm^2^/s) | 0.666±0.069 | 0.673±0.065 | 0.889 |
| Dxassoc(×10^-3^mm^2^/s) | 0.778±0.101 | 0.742±0.121 | 0.380 |
| Dxsubc(×10^-3^mm^2^/s) | 0.961±0.099 | 0.957±0.139 | 0.889 |
| Dyproj(×10^-3^mm^2^/s) | 0.455±0.069 | 0.391±0.055 | **0.018** |
| Dyassoc(×10^-3^mm^2^/s) | 1.092±0.121 | 0.941±0.176 | **0.001** |
| Dysubc(×10^-3^mm^2^/s) | 0.831±0.127 | 0.836±0.098 | 0.889 |
| Dzproj(×10^-3^mm^2^/s) | 1.205±0.110 | 1.272±0.109 | 0.098 |
| Dzassoc(×10^-3^mm^2^/s) | 0.471±0.097 | 0.678±0.160 | **<0.001** |
| Dzsubc(×10^-3^mm^2^/s) | 0.711±0.130 | 0.727±0.107 | 0.889 |
| ALPS index | 1.57±0.17 | 1.37±0.16 | **<0.001** |

SMA,Spinal Muscular Atrophy;Dxproj, diffusivity along the xaxis in projection fiber area;Dxassoc, diffusivity along the x-axis in association fiber area; Dxsubc, diffusivity along the x-axis in subcortical fiber area; Dyproj, diffusivity along the y-axis in projection fiber area; Dyassoc, diffusivity along the y-axis in association fiber area; Dysubc, diffusivity along the y-axis in subcortical fiber area; Dzproj, diffusivity along the z-axis in projection fiber area; Dzassoc, diffusivity along the z-axis in association fiber area; Dzsubc, diffusivity along the z-axis in subcortical fiber area.

**False discovery rate-adjusted p-values less than 0.05 indicate statistical significance*

A


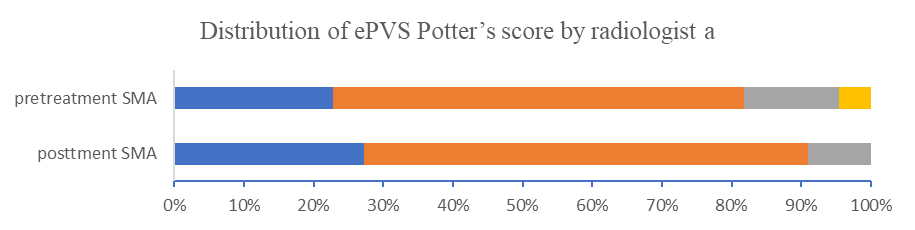


B


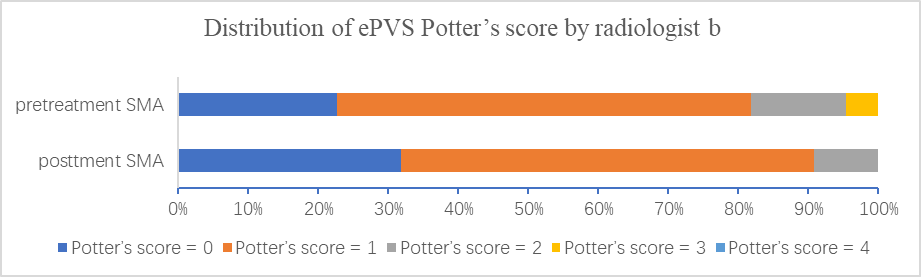


**Fig S1 Potter’s score distribution.** The bar plot represents the distribution of Potter’s score for ePVS by radiologist a (A) and radiologist b (B) in pretreatment and posttreatment SMA patients, bar width reflects the number of subjects in each group.


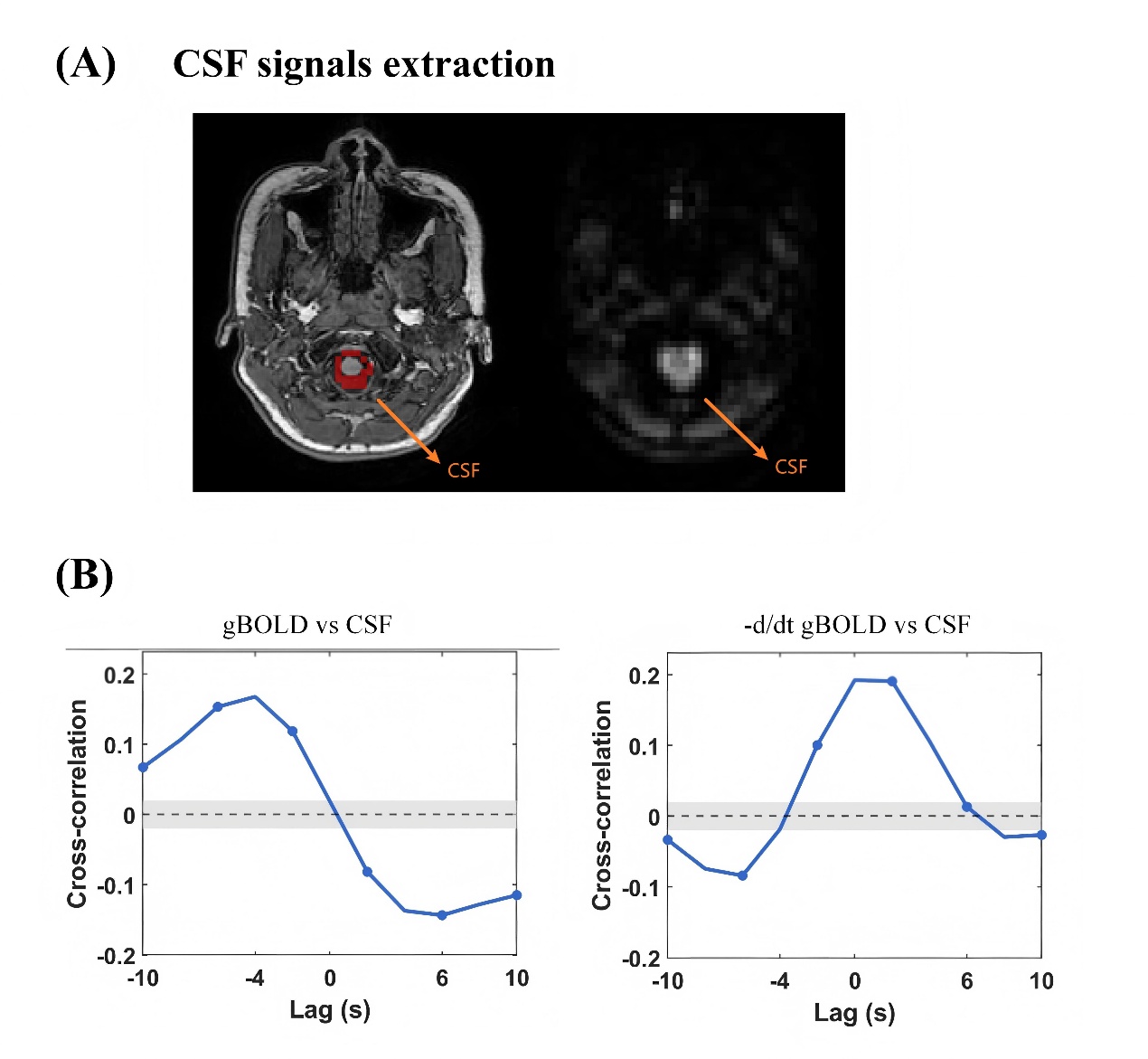


**Fig S2 （**A) The bottom slice of the axial T1W and rsfMRI images. The red area was used to extract the CSF signal. (B) The mean gBOLD-CSF crosscorrelation function (N = 46) was characterized by a positive peak (r = 0.168, p < 0.001; permutation test with n = 10,000) around a lag of −4 s and a negative peak (r = − 0.143, p < 0.001; permutation test with n = 10,000) at a lag of +6 s. The cross-correlation function between the CSF signal and the negative derivative of the gBOLD signal was also calculated, characterized by a large positive peak around a lag of 0s (d/dt r = -0.193, p < 0.001; permutation test of n =10,000). Gray dashed lines denote the 95% confidence interval of the null distribution. Abbreviation: gBOLD, global blood‑oxygen‑level‑dependent signal.
